# circALPL Sponges miR-127 to Promote Gastric Cancer Progression by Enhancing MTDH Expression

**DOI:** 10.7150/jca.49942

**Published:** 2021-06-11

**Authors:** Peng Wu, Dongmei Ye, Jiaoyan Li, Fei Yan, Xin Jin, Zhiwei Zhang, Zhenfa Li

**Affiliations:** 1 Affiliated Hengyang Hospital, Southern Medical University (Hengyang Central Hospital), 12# Yancheng road, Hengyang 421001, Hunan province, China.; 2 Department of Critical Care Medicine, Hengyang Maternal and Child Health Hospital, Hengyang, 421001, Hunan Province, China.; 3 Cancer Research Institute of Hengyang Medical College, University of South China; Key Laboratory of Cancer Cellular and Molecular Pathology of Hunan, 28 Changsheng Road, Hengyang, Hunan 421001, Hunan Province, China.

**Keywords:** circALPL, gastric cancer, MTDH, microRNA, competitive endogenous RNAs

## Abstract

**Background:** CircRNA plays an important role in cancer progression. However, the potential mechanism of circRNA in gastric cancer remains unknown. In this study, we aimed to investigate the specific mechanism of circALPL in gastric cancer.

**Methods**: Using a high-throughput microarray, we found that circALPL was upregulated in gastric cancer cell lines. RT-qPCR was used to measure the circALPL expression level in gastric cell lines and tissue. Transwell, CCK-8, and metastasis assays were performed to learn the function after circALPL was inhibited.

**Results**: circALPL downregulation suppresses the invasion and proliferation ability of gastric cancer cells. Additionally, the underlying pathway of circALPL was studied using luciferase reporter assays and RNA immunoprecipitation assays. The results showed that circALPL promotes gastric cancer progression by sponging miR-127, thus upregulating MTDH.

**Conclusion:** The circALPL-miR-127-MTDH pathway plays a vital role in gastric cancer proliferation and metastasis. circALPL might be a new therapeutic target in gastric cancer.

## Introduction

Gastric cancer (GC) is one of the most malignant tumors worldwide and has the fifth highest incidence and third highest mortality rate according to the 2015 Global Cancer Statistics Report 1. Although the incidence rates and mortality of gastric cancer gradually decrease with the use of proton pump inhibitors drugs and anti-HP antibodies, the five-year survival outcome of terminal gastric cancer patients is still very poor 2. Thus, finding sensitive and specific detection methods or biomarkers to improve the early detection rate of gastric cancer and reduce morbidity and mortality is urgent.

CircRNAs have received extensive attention in biomedicine and life sciences in recent years. As a novel endogenous noncoding RNA, circRNA is widely expressed in mammalian cells 3. circRNAs are created by the back splicing of exons or introns 4. The cyclic ring structure is highly converted. Because of their special structure, circRNAs can regulate the expression of key genes through a variety of integrated mechanisms, including sponge microRNAs (miRNAs) and coding proteins 5. CircRNAs play an important role in the process of many diseases, especially in cancer 6. Li et al. found that circITCH is significantly decreased in esophageal squamous cell carcinoma, and it might interact with miR-7, miR-17 and miR214 to upregulate ITCH expression, which would promote ubiquitination-mediated Dvl2 degradation and reduce the expression of oncogene c-myc 7. CircFBXW7 is downregulated in tumors and inhibits glioma cell proliferation and metastasis by encoding the species 21 kDA new protein FBXW7-185AA and blocking miR-197-3p 8. Although the biological importance of circRNAs is becoming increasingly clear, the mechanism of circRNA regulation is not fully understood.

In this study, we identified a new upregulated new circRNA, hsa-circ-0010519, in the gastric cancer cell line MGC-803 by analyzing the circRNA microarray results. circALPL was upregulated in gastric cancer tissues and GC cell lines. Knockdown of circALPL inhibited gastric cancer cell proliferation and invasion into the lung. However, it induced cancer cell apoptosis. Luciferase reporter assays and RNA immunoprecipitation assays were conducted to explain the potential mechanism of circALPL. In summary, our study revealed the vital role of the circALPL-miR-127- MTDH axis in gastric cancer progression.

## Method and materials

### Clinical sample data

Fifty pairs of cancer tissues and adjacent normal tissues were collected from patients aged 36-69 years old who were first diagnosed with gastric cancer and underwent surgery at the Hengyang Medical School of University of South China from 2016 to 2018. After surgery, the sectioned cancerous tissues and paired normal gastric tissues were immediately stored in RNAlater (Ambion). All patients provided written informed consent, and this study was approved by the Medical Ethics Committee of Hengyang Medical School of University of South China.

### Cell culture and transfection

Gastric cancer cell lines and the normal cell line GES-1 were purchased from the American Type Culture Collection (ATCC). Cell lines were cultured in DMEM (Gibco, Carlsbad, CA, USA) containing 10% FBS (Gibco) and maintained in a humidified incubator at 5% CO2. siRNAs (50 nM) were transfected into MGC-803 and SGC-7901 cells using Lipofectamine 3000 (Invitrogen) following the manufacturer's instructions.

### Cell Counting Kit-8 assay

si-circALPL sequences were transfected into cancer cells. Twenty-four hours later, 1X103 cancer cells were seeded into a 96-well plate. The plate was incubated for 24, 48, 72, and 96 h in the incubator. Ten microliters of CCK-8 solution was added to each plate well. Then, the cells were incubated for 1-4 h. The absorbance was measured at 450 nm using a microplate reader (Bio-Tek EPOCH2, Vermont, USA).

### Transwell assay

Transwell assays were conducted in transwell chambers and 24-well plates. The chamber was placed in the plate. A total of 1 x 10^5^ cells were centrifuged in 200 µl FBS-free DMEM and then added to the upper transwell compartments. Then, 500 µl of DMEM containing 10% FBS was added to the lower chamber. The transwell plate was incubated for 24 h. The medium in the chamber was removed, and the upper layer of cells was swabbed. The cells in the lower compartment of the chamber were fixed with 4% formaldehyde solution for 10 min. The cells were stained with 0.1% crystal violet for 15 min. Remaining stain was removed by washing with PBS. The number of invading cells was counted under a NIKON ECLIPSE 80i microscope (Nikon Instruments, NY, USA).

### RNA immunoprecipitation (RIP)

Cells were cotransfected with MS2bs-circALPL, MS2bs-circALPLmt or MS2bs-Rluc. Forty-eight hours later, the experiment was conducted with a Magna RIP RNA-Binding Protein Immunoprecipitation Kit (Millipore). The abundance of miR-127 was detected after purifying the RNA complexes. The RIP assay based on Ago2 was conducted with an anti-Ago2 antibody (Millipore). The enrichment of circALPL, MTDH and miR-127 was quantified after purification.

### Apoptosis assay

si-circCTR, si-circALPL-1 or si-circALPL-2 were transfected into MGC-803 and SGC-7901 cells. Annexin V-FITC staining and flow cytometry were performed according to the Annexin V-fluorescein Isothiocynate Apoptosis Detection Kit guidelines (KeyGen, Nanjing, China). Briefly, 1x10^5^ cells were washed with cold PBS, resuspended in 300 µL of 1X binding buffer and added to 5 µL of Annexin V-FITC and then incubated for 15 min in the dark at room temperature. The samples were mixed with 5 µL propidium iodide before analysis. A FACS Calibur (Becton Dickinson, San Jose, CA) was used to perform flow cytometric analysis immediately.

### Luciferase activity assay

Before transfection, 1x105 cells per well were plated in 24-well plates for 24 h. The wild-type or mutant vectors (200 ng) were transfected into cells together with miR-127-5p mimics (200 pmol) or control using Lipofectamine 3000 (Invitrogen) according to the manufacturer's protocol. Luciferase activity was measured 24 h after transfection using the Dual Luciferase Reporter Assay System (Promega).

### *In vivo* lung metastasis model

For the lung metastasis assay, cells (1×10^5^) were injected through the tail vein (five mice per group). Then, the medium containing si-circCTR, si-circALPL#1 or si-circALPL#1 was injected into the nude mice via the tail vein. The lungs were excised 8 weeks later, and the number of metastatic nodules was counted and validated via microscopy of hematoxylin and eosin (HE)-stained sections.

### RT-qPCR analysis

TRIzol (Invitrogen) was used to extract total RNA. Isolation of the nuclear and cytoplasmic components of cellular RNA was performed using NE-PER Nuclear and Cytoplasmic Extraction Reagents (Thermo Scientific). qRT-PCR assays were performed based on the manufacturer's guidelines for SYBR Premix Ex Taq™ (Takara, Japan) and the All-in-One™ miRNA qRT-PCR Detection Kit (GeneCopoeia). All primers were acquired from Sangon Biotech (Shanghai, China). All primers are listed in the supplemental material.

### Western blot analysis

The cells were collected, and the remaining FBS was removed. The total protein in cells was lysed with RIPA:PMSF (100:1). The proteins were boiled at 100°C for 10 min. A total of 20-30 μg of protein was loaded into each well in the SDS-PAGE gel and run for 2 h at 100 V. Then, the protein was transferred to a PVDF membrane in transfer buffer for 2 h at 200 mA. The nonspecific antigen was blocked using 5% skim milk for 1 h at room temperature. The membrane was incubated with a 1:3000 dilution of primary antibody (MTDH, 1:3000, Abcam) overnight at 4°C and then incubated with the secondary antibody at room temperature for 1 h. The protein bands were visualized in a ChemiImager System.

### Statistics

All data are shown as the mean ± SD. The differences between two unpaired groups were analyzed using Student's two-tailed unpaired t-test. A *P* value <0.05 was considered statistically significant.

## Results

### circALPL is upregulated in gastric cancer

**Figure 1:**
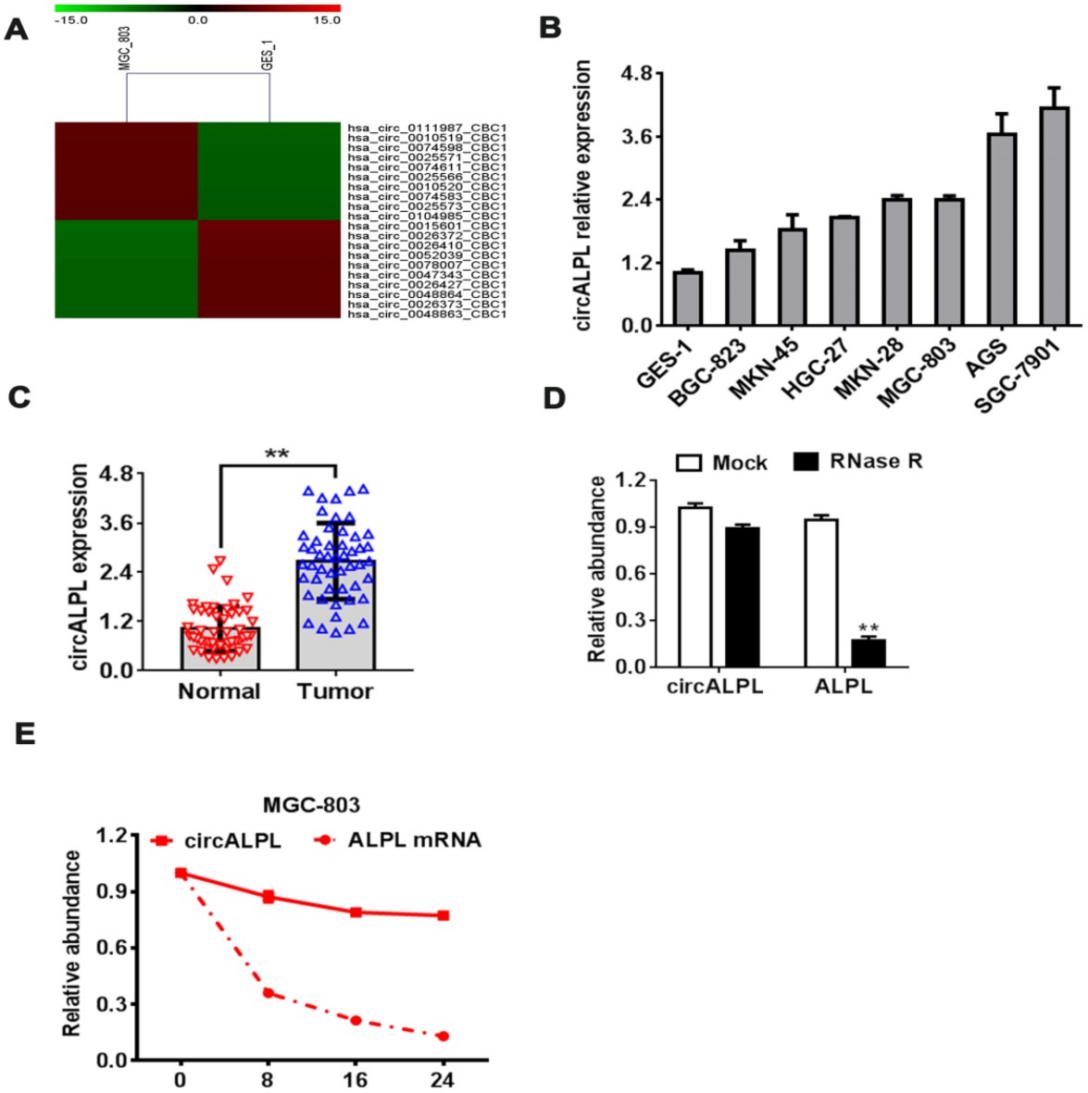
** circALPL upregulated in GC.** (A) The top 10 upregulated and downregulated circRNAs in the gastric cancer cell line microarray. (B) qRT-PCR was conducted to confirm the expression levels of circALPL in human gastric mucosal epithelial cell (GES-1) and gastric cancer cell lines. (C) The expression level of circALPL in normal gastric tissue and tumor tissue. (D) The circular structure of circALPL was confirmed by the RNase R assay. (E) circALPL is more stable than ALPL linear mRNA over time. A *P* value <0.05 was considered significant. **, *P*<0.01.

To explore the function of circRNA in GC, we performed a circRNA microarray using the gastric cancer cell lines MGC-803 and human gastric mucosal epithelial cell (GES-1). The analysis results showed the top 10 upregulated and downregulated circRNAs. hsa-circ-0010519 is one of the most upregulated circRNAs in the gastric cancer cell line (Figure 1A). Based on the derived gene of hsa-circ-0010519, we designated hsa-circ-0010519 as circALPL. We found the same upregulation phenomenon in other gastric cancer cell lines and cancer tissues (Figure 1B-C). To confirm the RNase R resistance characteristic of circALPL, we conducted an RNase R digestion experiment to compare the stability of circALPL and its derived gene ALPL linear mRNA. We found that circALPL could not be digested by RNase R. In contrast, linear ALPL mRNA was digested in RNase R (Figure 1D), and the abundance of ALPL mRNA decreased over time (Figure 1E). circALPL is more stable than linear RNA. Considering all the results above, we can conclude that circALPL is a circRNA and is upregulated in gastric cancer.

### Decreased circALPL expression can inhibit gastric cancer proliferation by inducing apoptosis

**Figure 2:**
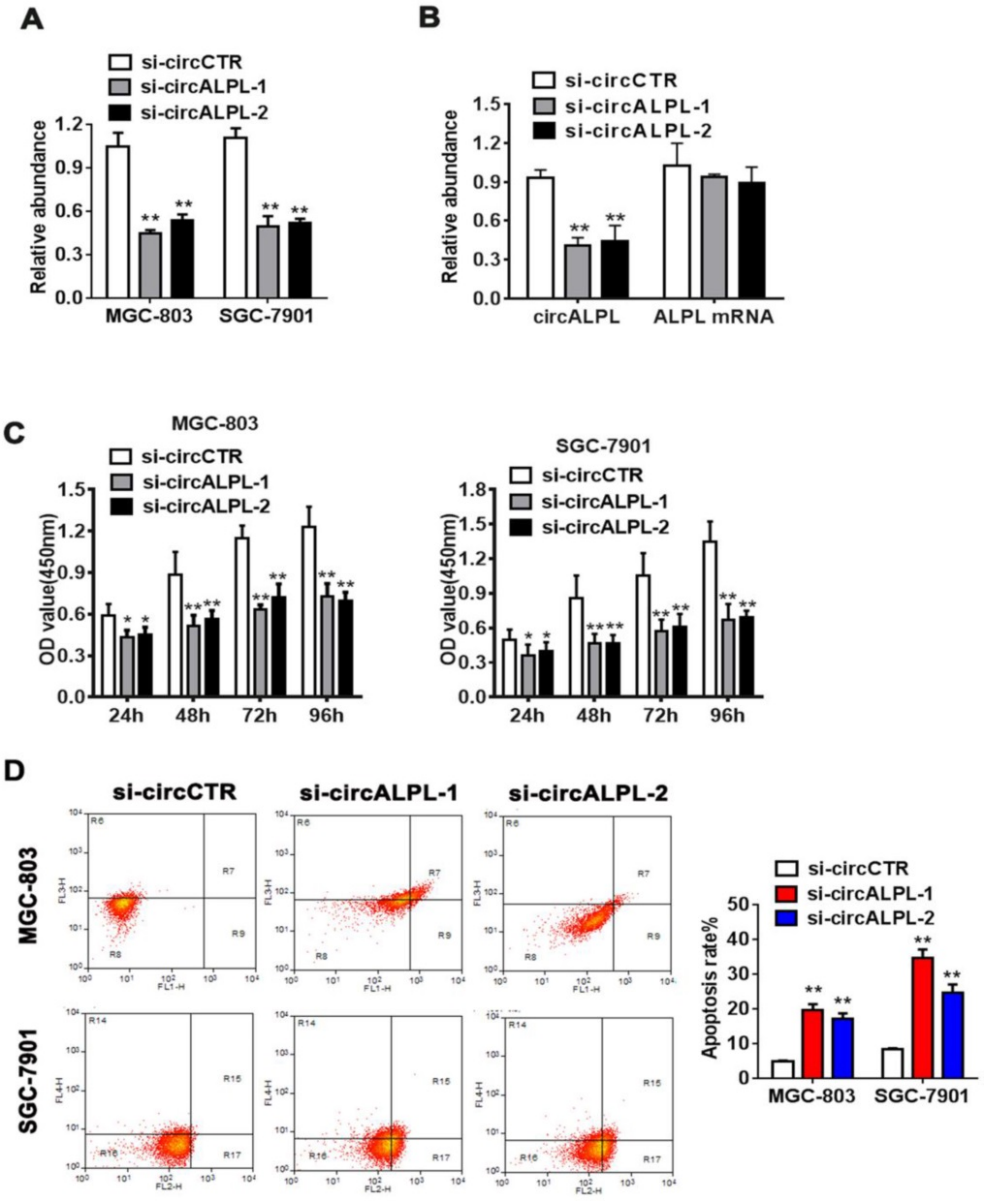
** circALPL knockdown inhibited the proliferation and increased the apoptosis ratio of GC cells.** (A) Two RNA interference sequences of circALPL successfully suppressed circALPL expression. (B) Two kinds of si-circALPL sequences did not affect ALPL mRNA expression. (C) A CCK-8 assay was conducted to detect the proliferation ability of gastric cancer cells when circALPL was inhibited over time. (D) An apoptosis assay was performed to determine the effect of circALPL deregulation on cell apoptosis. The left lower quadrant (FITC-/PI-) shows living cells, the right upper quadrant (FITC+/PI+) shows necrotic cells, and the right lower quadrant (FITC+/PI-) shows apoptotic cells. **, *P*<0.01.

To further study the mechanism of circALPL in GC proliferation, we used the RNA interference method to suppress circALPL expression. We designed two kinds of siRNA sequences. Then, the inhibitory effect was detected in the MGC-803 and SGC-7901 cell lines by qRT-PCR. The results showed that si-circALPL-1 and si-circALPL-2 successfully inhibited circALPL expression without decreasing the ALPL mRNA level (Figure 2A-B). CCK-8 and apoptosis assays were used to confirm the function of circALPL in cancer cells. Suppressing circALPL can inhibit cell proliferation (Figure 2C), but it increases the apoptosis ratio (Figure 2D).

### circALPL downregulation inhibits the metastatic ability of gastric cancer cells

**Figure 3:**
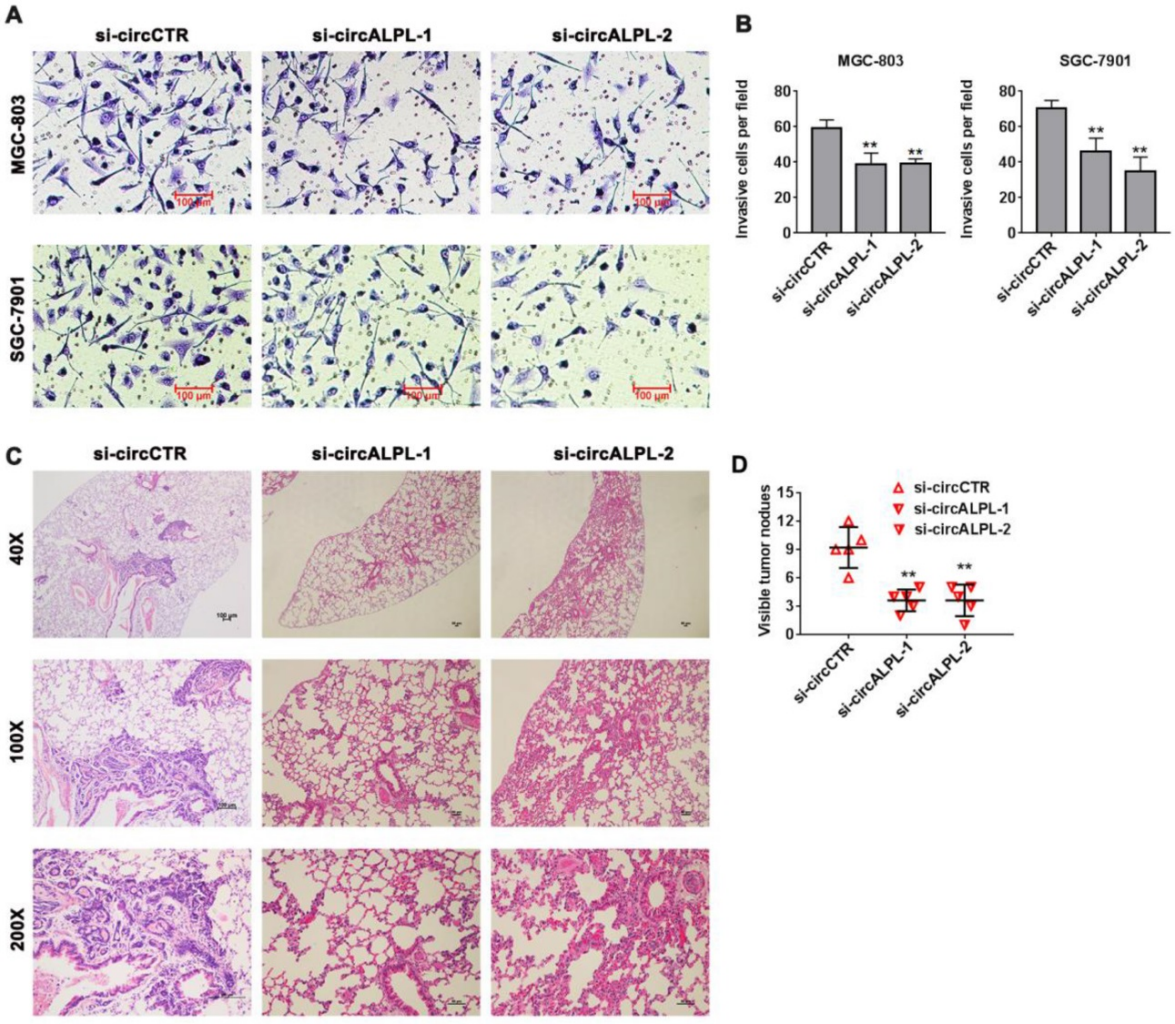
** Downregulation of circALPL inhibited gastric cancer cell invasion and tumor metastasis.** (A) The transwell assay was used to analyze the invasion ability of circALPL-inhibited GC cells. (B) The invasion cells per field were counted. **, *P*<0.01. (C) Representative HE images of metastatic nodule sections from an *in vivo* lung metastasis model. (D) The numbers of lung metastasis nodules were counted. **, *P*<0.01.

Transwell assays were carried out to estimate the effect of circALPL on the metastatic ability of GC. After circALPL knockdown, the cancer cell metastasis ability was suppressed (Figure 3A-B). To further confirm the function of circALPL in metastasis *in vivo*, we established a lung metastasis model. The results demonstrated that downregulation of circALPL decreased the number of metastatic GC cells *in vivo* (Figure 3C-D). All evidence indicated that circALPL might play an important role in GC metastasis.

### circALPL is a ceRNA of miR-127-5p

**Figure 4:**
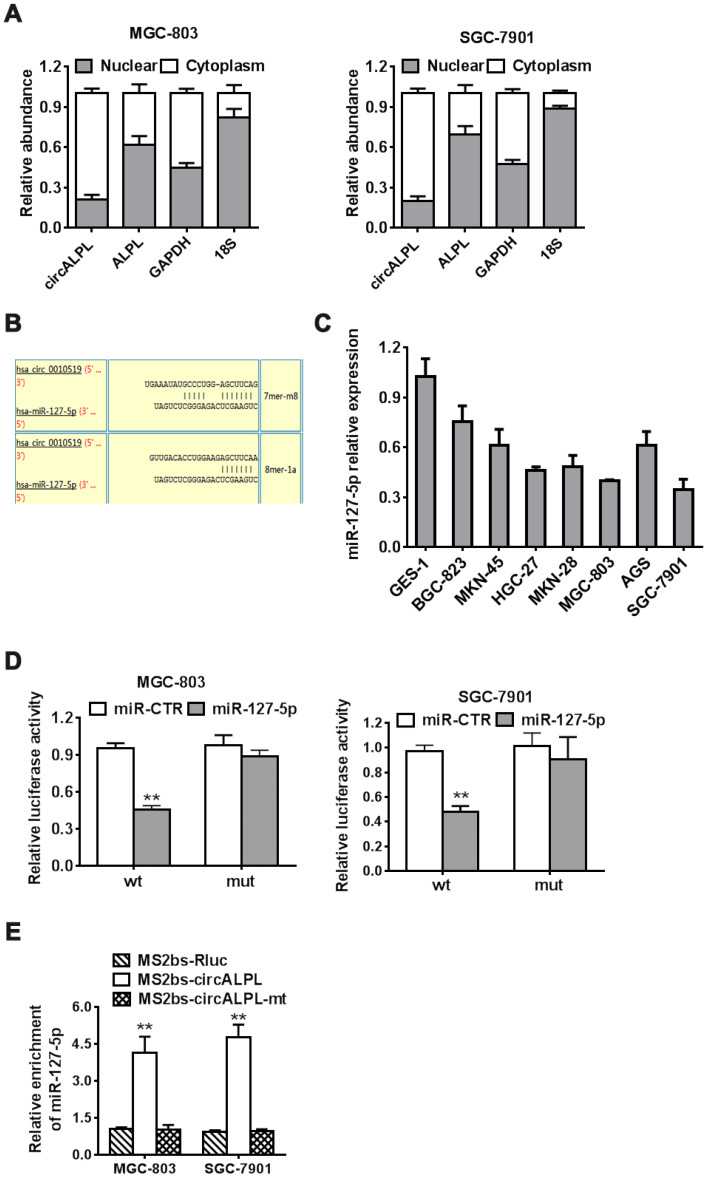
** circALPL acts as a sponge of miR-127-5p.** (A) qRT-PCR analysis of circALPL and ALPL in nuclear and cytoplasmic fractions. GAPDH acted as the cytoplasmic control. 18S was used as the nuclear control. (B) The predicted interacting site of mi-R-127-5p in circALPL. (C) The expression level of miR-127-5p in gastric cancer cell lines and GES-1. (D) Luciferase reporter assay of MGC-803 and SGC-7901 cell lines cotransfected with miR-127-5p mimics and circALPL wild-type or mutant luciferase reporter. (E) In the MS2-based RIP assay, MS2bs-circALPL, MS2bs-circALPL-mt or the control group was transfected into the MGC-803 and SGC-7901 cell lines. Then, we compared their miR-127-5p enrichment. **,* P*<0.01.

After removing the nuclear and cytoplasmic parts of cells, we then determined the abundance of circALPL and ALPL mRNA. circALPL predominately exists in the cytoplasm, which means that circALPL might act as a molecular sponge (Figure 4A). To elucidate the specific mechanism of circALPL in GC progression, we predicted the potential combination of the circALPL microRNA with the circular RNA interactome (https://circinteractome.nia.nih.gov). The prediction results showed that there are several binding sites between circALPL and miR-127-5p (Figure 4B). miR-127-5p is generally downregulated in gastric cancer cell lines (Figure 4C). According to the interaction site of circALPL and miR-127-5p, we designed wild-type and mutant circALPL reporters. The miR-127-5p mimic or miR-CTR were transfected together with a luciferase reporter. Luciferase activity was remarkably reduced when the wild-type circALPL reporter was transfected with miR-127-5p (Figure 4D). The results proved that circALPL can combine with miR-127-5p. To confirm the direct interaction of circALPL and miR-127-5p, we carried out RNA immunoprecipitation (RIP) assays. In both cancer cell lines, miR-127-5p was enriched in the MS2bs-circALPL group (Figure 4E). This demonstrated that circALPL can sponge miR-127-5p, thus modulating the deterioration of gastric cancer.

### circALPL promotes gastric cancer progression by the circALPL-miR-127-MTDH pathway

**Figure 5:**
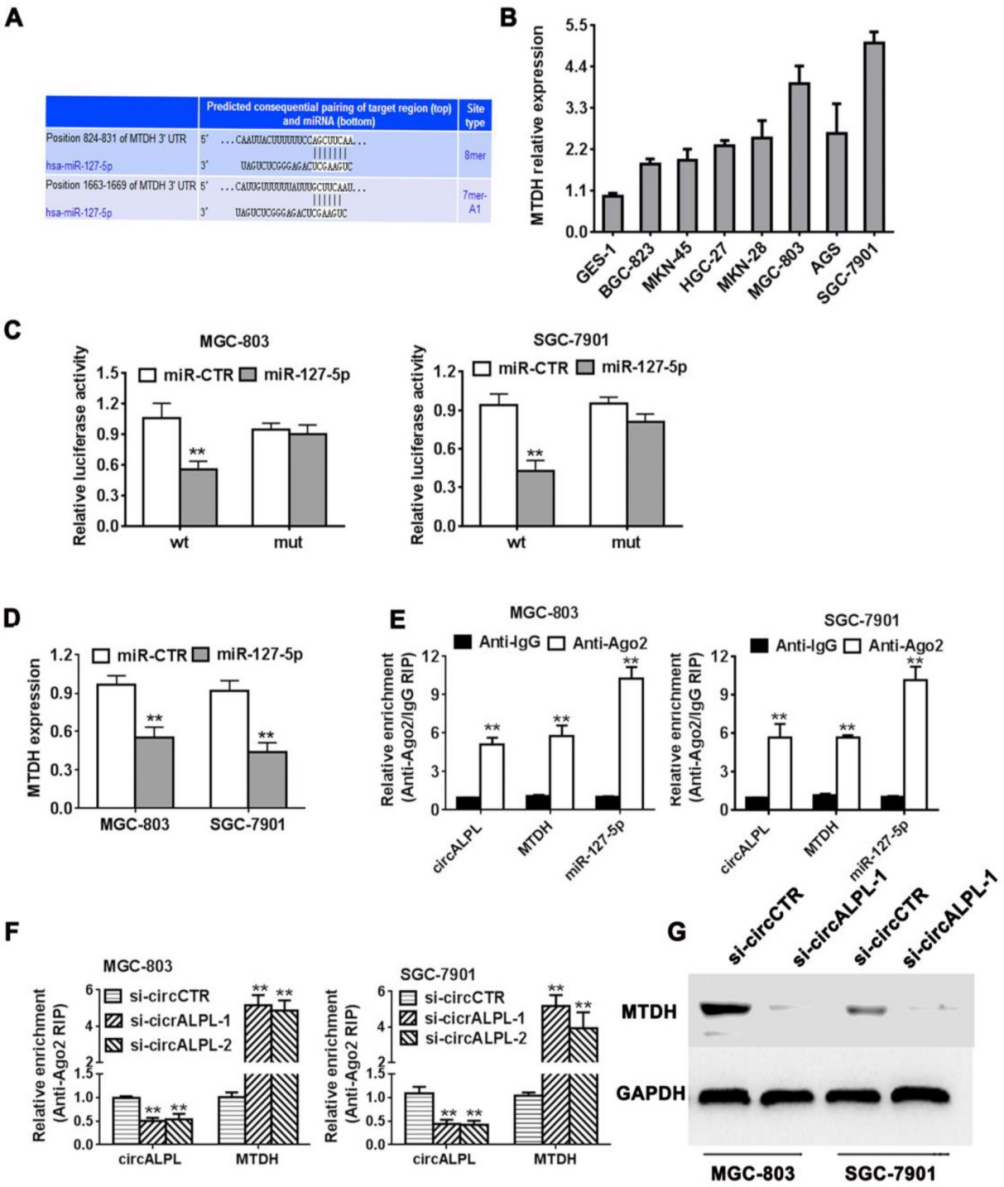
**circALPL promotes gastric cancer progression through the circALPL-miR-127-MTDH axis**. (A) The predicted binding sites of miR-127 in MTDH mRNA. (B) The expression level of MTDH in GC cell lines. (C) Luciferase reporter assay of MGC-803 and SGC-7901 cell lines cotransfected with miR-127-5p mimics and MTDH wild type or mutant luciferase reporter. (D) Expression of MTDH was decreased after overexpression of miR-127. (E) Enrichment of circALPL, MTDH and miR-127 on Ago2 assessed by RIP assay. (F) Enrichment of Ago2 to circALPL was decreased, while MTDH was increased after knockdown of circIQCH. (G) Western blot assay with the knockdown of circALPL resulted in a decrease in MTDH expression. **P<0.01.

We predicted the target gene of miR-127-5p using TargetScan (http://www.targetscan.org). We found that the MTDH gene shared an interaction site with miR-127-5p (Figure 5A). It has been reported that MTDH is an oncogene in some kinds of cancer. MTDH was also overexpressed in gastric cancer cell lines (Figure 5B). Next, we used the luciferase reporter assay to detect the interaction between miR-127 and MTDH. The results showed that the luciferase intensity decreased significantly when the MTDH wild-type reporter was cotransfected with the miR-127-4p mimic (Figure 5C). Additionally, increasing miR-127-5p expression led to a reduction in MTDH (Figure 5D). In summary, miR-127-5p can directly reduce MTDH expression. In addition, Ago2-related RIP assays demonstrated that circALPL, miR-127-5p and MTDH were all concentrated in the anti-Ago2 group compared to the anti-IgG group (Figure 5E). circALPL knockdown obviously increased the MTDH expression level (Figure 5F). Western blot analysis revealed that circALPL downregulation follows the decrease in MTDH (Figure 5G).

## Discussion

circRNAs are a new kind of ncRNA, and circRNAs have become a hot topic in the life science field and have attracted the attention of many researchers. With the considerable progress in high-throughput sequencing technology and bioinformatics algorithms, more circRNAs have been identified and studied 9. Recently, many circRNAs have been confirmed and well-studied in GC research. In most GC research on circRNAs, the numbers of upregulated and downregulated circRNAs are approximately equal 10. This result indicates that different circRNAs may play distinct roles in GC progression. Huang et al. found that the upregulation of circAKT3 is associated with poor outcomes in GC patients who were treated with cisplatin (CDDP) 11. circAKT3 enhanced DNA damage repair and suppressed apoptosis by sponging miR-198 and increasing the expression of the PIK3R1 gene. circFAT1 was downregulated in GC tissues and cells 12. circFAT1 sponged miR-548g and suppressed YBX1 protein expression in GC growth inhibition. Therefore, circRNA might serve as a potential suppressor and biomarker of GC. In this study, we conducted a circRNA high-throughput microarray and found that hsa-circ-0010519 (circALPL) was the most upregulated circRNA in gastric cancer cell lines. Similarly, circALPL increased in gastric cancer tissues. Inhibiting circALPL suppressed cancer cell proliferation and invasion into the lung *in vivo* and *in vitro*. However, the apoptosis ratio increased after circALPL decreased. To further investigate the molecular mechanism of circALPL, we predicted the downstream molecule of circALPL.

miRNA is considered the most well-known subclass of noncoding RNA and modulates target key gene expression through intercellular signaling in the tumor microenvironment 13. miRNA-127-5p was found to be involved in CpG island methylation 14 and lung development 15. miR-127 is downregulated in primary tumors and various cancer cell lines 16-18. miR-127 can downregulate BCL6 at the translational level 19. Thus, increased miR-127 expression in cancer cells may have an anticancer effect by inhibiting BCL6. Deregulation of miR-127 was found in breast cancer tissues, and low expression of miR-127 was correlated with worse clinical outcome in breast cancer 20. miR-127-5p also decreased in gastric cancer cell lines. The luciferase reporter and RNA immunoprecipitation assay results revealed that circALPL can promote gastric cancer progression by directly binding to miR-127-5p. MTDH was identified as a target gene of miR-127-5P.

Metadherin (MTDH), also named astrocyte elevated gene 1 (AEG-1), was first reported as a late response gene that was induced by HIV infection or TNF-α dysregulation in human fetal astrocytes 21. MTDH is regarded as an oncogene in multiple cancers and is usually correlated with poor outcomes 22-24. MTDH downregulates SOCS-1 expression to amplify NF-κB signaling. MTDH promotes inflammation in the tumor microenvironment in gastric cancer 25. In our study, MTDH was modulated by miR-127-5p with its binding site in the 3'-UTR. circALPL knockdown can inhibit the expression of MTDH, which means that circALPL can regulate MTDH levels.

In summary, our study revealed that the circALPL-miR-127-5p-MTDH pathway plays an important role in gastric proliferation and metastasis. circALPL might become a potential biomarker and therapeutic target of gastric cancer.

## Supplementary Material

Supplementary table.
