# Supplementary material for: circALPL Sponges miR-127 to Promote Gastric Cancer Progression by Enhancing MTDH Expression
Source: J Cancer. 2021 Jun 11;12(16):4924–32. doi: 10.7150/jca.49942 (PMC8247368; doi:10.7150/jca.49942)
Supplement: Supplementary file 1 — Supplementary table. [file jcav12p4924s1.pdf]

Supplementary Table 1. Primer sequences for qRT-PCRs used in this study

Construct	Direction	Sequence(5'-3')
circALPL	Forward	TGGGCTCCAGGGATAAAGCA
	Reverse	GCGGTTCCAGATGAAGTGGG
ALPL	Forward	ACCACCACGAGAGTGAACCA
	Reverse	CGTTGTCTGAGTACCAGTCCC
MTDH	Forward	AAATGGGCGGACTGTTGAAGT
	Reverse	CTGTTTTGCACTGCTTTAGCAT
18s	Forward	AACTGGAATCGCATCAGGAC
	Reverse	AGGAGCTGCTCTGGGTGTAA
GAPDH	Forward	GGAGCGAGATCCCTCCAAAAT
	Reverse	GGCTGTTGTCATACTTCTCATGG
